# Residues at the tip of the pore loop of NR3B-containing NMDA receptors determine Ca^2+ ^permeability and Mg^2+ ^block

**DOI:** 10.1186/1471-2202-11-133

**Published:** 2010-10-19

**Authors:** Nora A Cavara, Angela Orth, Gordon Hicking, Guiscard Seebohm, Michael Hollmann

**Affiliations:** 1 Department of Biochemistry I - Receptor Biochemistry, Ruhr University Bochum, Universitaetsstrasse 150, D-44780 Bochum, Germany; 2 International Graduate School of Neuroscience (IGSN), Ruhr University Bochum, Bochum, Germany; 3 Ruhr University Research School, Ruhr University Bochum, Bochum, Germany; 4 DFG Graduate School 736 "Development and Plasticity of the Nervous System: Molecular, synaptic and cellular mechanisms", Ruhr University Bochum, Bochum, Germany

## Abstract

**Background:**

Members of the complex N-methyl-D-aspartate receptor (NMDAR) subfamily of ionotropic glutamate receptors (iGluRs) conventionally assemble from NR1 and NR2 subunits, the composition of which determines receptor properties. Hallmark features of conventional NMDARs include the requirement for a coagonist, voltage-dependent block by Mg^2+^, and high permeability for Ca^2+^. Both Mg^2+ ^sensitivity and Ca^2+ ^permeability are critically dependent on the amino acids at the N and N+1 positions of NR1 and NR2. The recently discovered NR3 subunits feature an unprecedented glycine-arginine combination at those critical sites within the pore. Diheteromers assembled from NR1 and NR3 are not blocked by Mg^2+ ^and are not permeable for Ca^2+^.

**Results:**

Employing site-directed mutagenesis of receptor subunits, electrophysiological characterization of mutants in a heterologous expression system, and molecular modeling of the NMDAR pore region, we have investigated the contribution of the unusual NR3 N and N+1 site residues to the unique functional characteristics of receptors containing these subunits. Contrary to previous studies, we provide evidence that both the NR3 N and N+1 site amino acids are critically involved in mediating the unique pore properties. Ca^2+ ^permeability could be rescued by mutating the NR3 N site glycine to the NR1-like asparagine. Voltage-dependent Mg^2+ ^block could be established by providing an Mg^2+ ^coordination site at either the NR3 N or N+1 positions. Conversely, "conventional" receptors assembled from NR1 and NR2 could be made Mg^2+ ^insensitive and Ca^2+ ^impermeable by equipping either subunit with the NR3-like glycine at their N positions, with a stronger contribution of the NR1 subunit.

**Conclusions:**

This study sheds light on the structure-function relationship of the least characterized member of the NMDAR subfamily. Contrary to previous reports, we provide evidence for a critical functional involvement of the NR3 N and N+1 site amino acids, and propose them to be the essential determinants for the unique pore properties mediated by this subunit.

## Background

Excitatory neurotransmission in the vertebrate central nervous system (CNS) is mediated to a large extent by ionotropic glutamate receptors (iGluRs). Signal transmission effected via the subfamily of N-methyl-D-aspartate (NMDA) receptors underlies complex long-term processes and accounts for the establishment of synaptic plasticity.

NMDA receptors (NMDARs) conventionally assemble as heterotetramers from NR1 and NR2 subunits, which carry the ligand-binding sites for glycine and glutamate, respectively. A characteristic of conventional NMDARs is their sensitivity to Mg^2+ ^ions. At membrane potentials below -40 mV, the ion channel of these receptors is blocked by extracellular Mg^2+ ^binding within the pore at the so-called N and N+1 positions [1-3].

The N position is located at the narrow constriction of the pore loop; it is the functional equivalent to the Q/R editing site in AMPA receptors. In both rodents and man, NR1 features an asparagine (Asn, N) at this position, followed by serine (Ser, S) [4,5]. The NR2 subunit has two adjacent asparagines at the N and N+1 positions. Thus, in conventional NMDARs, four Asn residues per pore form a constriction. The importance of this site for coordinating Mg^2+ ^has been recognized early on; experiments with mutant subunits demonstrated that Mg^2+ ^blockage critically depends on the amino acids at the N and N+1 positions [2,3,6].

The residues at the N and N+1 positions are also largely - but not exclusively - responsible for the characteristically high Ca^2+ ^permeability of conventional NMDARs [1-3].

With the discovery of the glycine-binding NR3 subunits [7,8], the question of the contribution of these subunits to NMDAR function arose. Strikingly, the N site of the NR3 subunits is unique in the NMDAR subfamily: In both NR3A and NR3B this position in rodents is occupied by glycine (Gly, G), followed by arginine (Arg, R) [7-9]. Even more exceptional is the human NR3B subunit, which features two adjacent arginines at the N and N+1 positions [9,10]. These unprecedented combinations of amino acids occupying those critical sites might effect dramatically altered NMDAR properties, particularly concerning Mg^2+ ^block and Ca^2+ ^permeability, when NR3 is present. In line with this, studies of heterologously expressed NMDARs revealed a reduction of Ca^2+ ^permeability in the presence of NR3B [11,12]. However, a definite influence of NR3 on the Mg^2+ ^sensitivity of conventional NMDARs is controversial: While several studies suggested no influence of NR3 on Mg^2+ ^sensitivity [8,9], a reduction of Mg^2+ ^block has been shown in heterologous expression systems [13] and NR3A-transgenic [14] and NR3A knockout mice [13].

The exact contribution of NR3 to the hallmark properties of NMDARs thus remains a matter awaiting clarification. A powerful tool to investigate the influence of NR3 presented itself with the discovery of excitatory glycine receptors built from NR1 and NR3 subunits in the absence of NR2 [15]. These NR1/NR3 diheteromers are fully activated by glycine alone, insensitive to Mg^2+^, and not permeable for Ca^2+ ^[15]. Whether they exist *in vivo *remains controversial, but their formation in heterologous expression systems can be exploited to understand the mechanisms by which the still poorly understood NR3 subunits influence the NMDAR core characteristics of Mg^2+ ^block and Ca^2+ ^permeability. Several studies have targeted the NR3 N site residue as a functional hotspot concerning Mg^2+ ^sensitivity and permeability for Ca^2+^, using site-directed mutagenesis of the N site amino acid and electrophysiological characterization of mutant subunits to clarify the functional impact of the NR3 N and N+1 site amino acids [7,8,12]. None of these studies reported a functional consequence of the NR3 N and N+1 site residues; however, in all cases investigations had been limited to triple expression experiments of NR1/NR2/NR3 receptors. In such an experimental design, the contribution of NR3-containing NMDARs might be masked by the additional assembly of conventional NR1/NR2 receptors. We have taken advantage of the fact that NR1 and NR3 assemble into diheteromers. To shed light on the mechanisms by which NR3 governs the unique properties of NR1/NR3 receptors, we have performed site-directed mutagenesis of NR1, NR2 and NR3 N and N+1 site residues. Mutant subunits were heterologously expressed in *Xenopus *oocytes and electrophysiologically characterized. Contrary to previous studies, we report a significant influence of the NR3 N and N+1 site amino acids on the ion channel properties, and suggest a mechanism by which NR3 may affect Mg^2+ ^sensitivity of NMDARs.

## Methods

### Accession numbers

The following clones were used: NR1-3a (GenBank: U08265), NR2A (GenBank: NM_012573), NR2B (GenBank: U11419), and NR3B (GenBank: NM_130455).

### Molecular biology

For expression in oocytes, all wild types and mutants were inserted into the vector pSGEM, a modified version of pGEMHE [17]. All N and N+1 site mutants were generated via PCR-directed mutagenesis using specific mismatch primers (microsynth.ch, Switzerland).

### cRNA synthesis

cRNA was synthesized from 1 μg of linearized DNA using an *in vitro *transcription kit (mMESSAGE mMACHINE™ Kit, Ambion) with an extended reaction time of 2 h with T7 polymerase.

### Electrophysiological measurements in *Xenopus laevis *oocytes

Oocytes of *Xenopus laevis *frogs (Nasco, Fort Atkinson, WI) were surgically removed from the ovaries and defolliculated as described previously [18]. Oocytes of stages V-VI were selected and maintained in Barth's solution supplemented with 100 μg/ml gentamicin, 40 μg/ml streptomycin, and 63 μg/ml penicillin. Injection of cRNA was performed using a nanoliter injector (World Precision Instruments, Sarasota, FL). 6 pmol of cRNA were injected for each receptor subunit (NR1 to NR3 ratio: 1:1; NR1 to NR2 ratio: 1:1; both cRNAs were mixed before injection in a standard volume). Four to six days after injection, oocyte current responses were recorded under voltage clamp at -70 mV holding potential with a TurboTec 10CX amplifier (npi Electronic, Tamm, Germany) controlled by Pulse software (HEKA Elektronik, Lambrecht, Germany). Recording pipettes were pulled from borosilicate glass (Hilgenberg, Malsfeld, Germany). Voltage electrodes had resistances of 0.5-1 MΩ and were filled with 3 M KCl; current electrodes had resistances of 0.5-1 MΩ and were filled with 3 M CsCl.

Agonists (100 μM glutamate, 10 μM glycine) and antagonists (0.5 mM Mg^2+^) were prepared in normal frog Ringer's solution (NFR) (115 mM NaCl, 2.5 mM KCl, 1.8 mM CaCl_2_, and 10 mM HEPES-NaOH, pH 7.2) for the determination of current responses and Mg^2+ ^block and were applied for 20 s by superfusion at a flow rate of 5 ml/min. Current-voltage relationships were determined by ramping holding potentials between -150 mV and +50 mV within 2 s in the presence of agonist and correcting them for background conductivities by subtracting the average of two control current-voltage relationships measured before and after agonist application. To limit a possible influence of Ca^2+^-activated Cl^- ^channels, oocytes were injected with 50 nl of 200 mM EGTA-NaOH, pH 8.0, 15 min prior to recordings of Mg^2+ ^sensitivities. For better comparison between different combinations, current-voltage relationships from 3-4 experiments were independently normalized to +20 mV and averaged.

To quantify Ca^2+ ^permeability, the conductance ratio of Ca^2+ ^to monovalent cations has to be calculated (P_Ca_^2+^/P_mono_). Therefore, reversal potentials were recorded in the presence of Ca^2+ ^as the sole putatively permeable ion. To inhibit any effect from endogenous Ca^2+^-activated chloride channels, oocytes were injected with 50 nl of 200 mM EGTA-NaOH, pH 8.0, 15 min prior to recording. Reversal potentials were determined from current-voltage (IV) relations recorded in 4 mM Ca^2+ ^Ringer's solution (CaR) (108.6 mM of the impermeable N-methyl-D-glucamine (NMDG), 4 mM CaCl_2_, 10 mM HEPES, pH 7.2 with 1 M HCl) and 8 mM CaR (114.2 mM NMDG, 8 mM CaCl_2_, 10 mM HEPES, pH 7.2 with 1 M HCl). If Ca^2+ ^ions are the only permeable ions on the outside, and if the cell is assumed to contain only monovalent cations with equal permeabilities on the inside, the Goldman-Hodgkin-Katz equation can be modified to include calcium as follows [19]:

$$\frac{P_{Ca^{2+}}}{P_{Mono}}=\frac{{\left[Mono\right]}_{i}\cdot e^{E_{rev}\cdot \frac{F}{RT}}\cdot (e^{E_{rev}\cdot \frac{F}{RT}}+1)}{{\left[Ca^{2+}\right]}_{O}\cdot 4}$$

with [Mono^+^]_i _= concentration of intracellular monovalent cations [mM], [Ca^2+^]_o _= concentration of extracellular calcium cations [mM], E_rev _= reversal potential [mV], F = Faraday constant [9.65 10^4 ^Cmol^-1^], R = gas constant [8.314 Jg^-1^K^-1^], T = temperature [K].

To determine the concentration of intracellular cations [Mono^+^]_i_, oocytes expressing the GluR6(Q) receptor with a known P_Ca_^2+^/P_mono _[20] were measured for each batch. Measurements of GluR6(Q) required pre-treatment of oocytes with concanavalin A for 8 min prior to recordings to inhibit desensitization. All reversal potentials were corrected for the junction potential of -4.5 mV.

For all combinations containing NR2, solutions were used at pH 7.2 as described above. All combinations featuring NR3 were measured at pH 8.0 to limit NR3-induced proton inhibition of current responses [21].

Data presented here are reported as mean ± SEM. Statistical significance was determined with an unpaired Student's t test.

### Homology modeling and molecular dynamics simulations

NR1-3a and NR3B homology models were created based on the KcsA structure (1k4c) Several individual modeling steps using YASARA Structure (version 9.10.5) were required [22]. The consensus homology models are based on the amino acid sequence and refinement of a high-resolution model using a CASP approved protocol [23]. The PDB-data base was searched for known structures with a similar sequence using PSI-BLAST [22] to identify potential modeling templates. In the case of NR1-3a and NR3B several fold recognition servers and knowledge-based manipulations (see below) had to be consulted and incorporated as well. The templates were ranked based on the alignment score and the structural quality according to WHAT_CHECK [24] obtained from the PDBFinder2 database [25]. Models were built for the top scoring templates. For each available template KcsA the alignment with the target sequence was iteratively optimized using the evolutionary information contained in related sequences (SwissProt and TrEMBL), the structural information contained in the template and the predicted target secondary structure [26] to obtain a structure-based alignment correction (partly based on SSALN scoring matrices) [27]. An indexed version of the PDB was used to determine the optimal loop anchor points and collect possible loop conformations if insertions and deletions and dead-end elimination was used to find an initial rotamer solution in the context of a simple repulsive energy function [28]. The loops were optimized by trying hundreds of different conformations, re-optimizing the side-chains for all of them. Fine-tuning of side-chain rotamers was performed considering electrostatic and knowledge-based packing interactions as well as solvation effects. An unrestrained high-resolution refinement with explicit solvent molecules was run using the AMBER03 force field and the result was validated to ensure that the refinement did not move the model in the wrong direction. A final hybrid model was built; bad regions in the top-scoring model were iteratively replaced with corresponding fragments from the other models. The transmembrane domain TM3 was modeled as an α-helix and docked to the outer face of the consensus homology pore module and 1 ns MD simulations were performed on the NR1-3a and NR3B homology model including transmembrane domain TM3 using YASARA with force field YASARA2 in default settings. This force field is very well suited for protein structure calculations in an H_2_O-filled box [29]. For all further calculations the AMBER03 force field was used with boundary periodic long-range Coulomb cutoff 7.86 Å [30]. The AMBER03 force field is an optimized for point-charge force field for molecular mechanics simulations of proteins [30]. The simulation cell was set at least 5Å around the atoms of the protein. Only, the pore helices and the selectivity filters were kept flexible, whereas the rest of the models were kept fixed. The fixed protein regions provided a surrounding for the flexible pore helices/selectivity filters. The outermost potassium ion in the selectivity filter of KcsA (S1) was substituted by an Mg^2+ ^ion. The parameters of the Mg^2+ ^ion were derived from the AMBER03 force field. The second potassium ion (S2) was replaced by a water molecule. The potassium ions in the outer pore region (S0) and in the lower selectivity filter (S3, S4) were replaced by sodium ions. The simulation box was filled with water at a density of 1.0 g/cm^3 ^(WaterDensity 1.0). The time step was set to 1.0 fs. Intermolecular forces were calculated every 2 simulation substeps. The simulations were performed for about 1 ns.

The coordination of the Mg^2+ ^ion was performed using the AMBER03 force field. The Mg^2+ ^ion was placed into the center of a water-filled box. The minimal distance between the Mg^2+ ^ion and the box walls distance was at least 6 Å. The simulations were performed for 1 nsec. The Mg^2+ ^ion stably coordinated six H_2_O molecules.

## Results

To examine the contribution of the N site to the unique properties of NR3-containing receptors, the amino acids at the N site and the adjacent position in the NR3B subunit were substituted for NR1-typical residues via site-directed mutagenesis. Conversely, NR1 and NR2 subunits were equipped with NR3-typical glycine residues at their N positions, to test if they, consequentially, might behave like NR3. All N site mutants were transcribed into cRNA, expressed in various combinations in *Xenopus laevis *oocytes, and examined electrophysiologically for hallmark NMDAR characteristics: agonist sensitivity, Mg^2+ ^block, and Ca^2+ ^permeability.

A simplified nomenclature for mutant subunits is employed: Residues at the N and N+1 site are named explicitly after the subunit's name. Thus, NR3B(NS) refers to an NR3B subunit with an asparagine at the N position, followed by serine.

In generating mutant subunits, special emphasis was placed on NR3, and a number of mutants were generated for this subunit: Firstly, as has been done previously [7,8], the N site glycine was mutated to the NMDAR-like residue asparagine (NR3B(NR)). As the N+1 position is occupied by arginine in NR3, and arginine at the N site equivalent of AMPARs is responsible for the very low Ca^2+ ^permeability of receptors including such a subunit, the NR3 N+1 arginine was mutated to asparagine (NR3B(GN)). To give the NR3 subunit a fully NR1-like N and N+1 site, the double mutant NR3B(NS) was generated.

Countermutants of NR1 and NR2B were equipped with the NR3-like glycine at their N position (NR1-3a(GS), NR2B(GN)).

### N and N+1 Site Mutants Assemble Functionally in a Heterologous Expression System

The exchange of a critical amino acid in the pore region might interfere with channel integrity and thus the subunit's function. Previous studies on NR1 and NR2 N site mutants reported an exchange to be possible without a loss of function [3,6], and several studies described the successful mutation of the NR3 N site [7,8,16]. In a first line of experiments, the general function of the mutated subunits was tested to see if those results could be corroborated.

**Figure 1:**
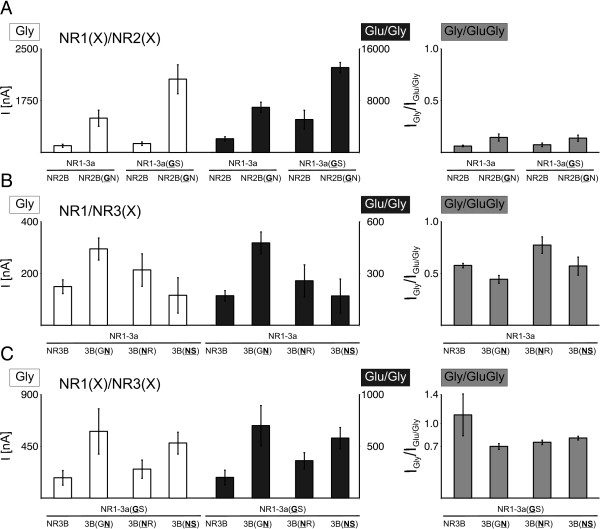
**Agonist-induced current responses of coexpressed mutant NMDAR subunits**. Mean current responses elicited by application of glycine alone (Gly, 10 μM) or together with 100μM glutamate (Glu) for NR1/NR2 and NR1/NR3 diheteromers assembled from wild type or mutant subunits. White bars represent mean amplitudes after the application of glycine, black bars denote mean responses after the coapplication of glutamate/glycine; all current amplitudes are shown in nA. The right diagram depicts the ratio of glycine- to glutamate/glycine-induced current responses for each combination. Data shown here are mean values ± SEM, n = 11-48 for NR1/NR2 combinations, n = 5-45 for NR1/NR3 diheteromers; *p < 0.05; **p < 0.01; ***p < 0.005 (Student's t-test).

As shown in Figure 1A (left diagram), mutation of the N site of either NR1 or NR2 to glycine did not interfere with the general ability of the subunit to assemble functionally: Both NR1-3a(GS)/NR2B and NR1-3a/NR2B(GN) receptors gave robust current responses after the application of glycine (left axis), but required additional glutamate for maximal activation (right axis). Coexpressed NR1-3a(GS)/NR2B(GN) subunits also assembled into functional receptors, with mean glutamate/glycine-induced current responses of 13076 ± 803 nA. The ratio of glycine- to glutamate/glycine-induced responses ranged between 0.06 ± 0.01 for the wild type combination of NR1-3a/NR2B and 0.15 ± 0.03 for NR1-3a/NR2B(GN) receptors (Figure 1A, right diagram).

A different agonist profile was recorded when NR3 was present instead of NR2: Again, all combinations tested were functional, but were fully activated by glycine alone, as is the case for wild type NR1/NR3 diheteromers. Mean amplitudes are summarized in graphical form in Figure 1B. As shown, function and a wild-typical agonist profile were seen regardless of whether the NR3 N site, or N+1 position, or both, were exchanged for NR1-like asparagine residues.

The same was true when the NR1 subunit was equipped with the NR3-like glycine at its N position (Figure 1C). For all NR3-containing diheteromers, the ratio of glycine- to glutamate/glycine-induced currents ranged between 0.7 ± 0.05 (for NR1-3a/NR3B(GN) diheteromers) and 1.1 ± 0.3 (for NR1-3a(GS)/NR3B receptors), indicating that receptors were fully activated by glycine alone. It is noteworthy, though, that current amplitudes differed between wild type diheteromers and receptors containing mutants: Especially receptors containing a mutated NR2 subunit mediated larger current responses than wild type diheteromers. All current amplitudes shown in Figure 1 are summarized in Table 1.

**Table 1:** Current amplitudes and Mg^2+ ^block of coexpressed mutant NMDAR subunits

	**I**_**Gly **_**[nA] ± SEM (*n*)**	**I**_**Glu/Gly **_**[nA] ± SEM (*n*)**	**Mg**^**2+ **^**Block I**_**Gly **_**[%] ± SEM (*n*)**	**Mg**^**2+ **^**Block I**_**Glu/Gly **_**[%] ± SEM (*n*)**
NR1-3a/NR2B	164 ± 38 (*33*)	2122 ± 328 (*48*)	125 ± 10 (*20*)	88 ± 3 (*24*)

NR1-3a/NR2B(**G**N)	827 ± 195 (*11*)	6959 ± 808.0 (*11*)	97 ± 25 (*5*)	71 ± 8 (*7*)

NR1-3a(**G**S)/NR2B	216 ± 43 (*11*)	5080 ± 1448 (*12*)	82 ± 12 (*9*)	56 ± 6 (*8*)

NR1-3a(**G**S)/NR2B(**G**N)	1764 ± 348 (*11*)	13076 ± 803 (*11*)	57 ± 5 (*8*)	25 ± 9 (*8*)

NR1-3a/NR3B	149 ± 27 (*45*)	171 ± 30 (*45*)	3 ± 1 (*27*)	13 ± 4 (*15*)

NR1-3a/NR3B(**N**R)	213 ± 62 (*15*)	258 ± 93 (*15*)	34 ± 8 (*11*)	30 ± 4 (*11*)

NR1-3a/NR3B(G**N**)	294 ± 42 (*5*)	476 ± 63 (*5*)	38 ± 6 (*5*)	46 ± 4 (*5*)

NR1-3a/NR3B(**NS**)	116 ± 68 (*18*)	170 ± 98 (*18*)	8 ± 8 (*12*)	31 ± 14 (*15*)

NR1-3a(**G**S)/NR3B	176 ± 64 (*13*)	200 ± 70 (*13*)	-3 ± 6 (*9*)	-4 ± 10 (*10*)

NR1-3a(**G**S)/NR3B(**N**R)	251 ± 79 (*5*)	361 ± 78 (*5*)	-3 ± 2 (*5*)	6 ± 3 (*5*)

NR1-3a(**G**S)/NR3B(G**N**)	579 ± 196 (*5*)	698 ± 194 (*6*)	5 ± 4 (*5*)	7 ± 3 (*6*)

NR1-3a(**G**S)/NR3B(**NS**)	252 ± 79 (*5*)	361 ± 78 (*5*)	-3 ± 2 (*5*)	6 ± 3 (*5*)

Overview of all current amplitudes and Mg^2+ ^blocks as shown in Figs. 1 and 4. Current responses are mediated by diheteromers featuring N and N+1 site mutant NMDAR subunits measured upon activation by either glycine alone (Gly, 10 μM) or by Gly in addition to glutamate (Glu, 100 μM). Values for current responses are given in nA ± SEM (standard error of the mean). The Mg^2+ ^block is given as % of blocked steady-state current ± SEM. n = number of cells.

As function and a wild type-consistent agonist profile were established for all mutated subunits, additional measurements concerning the NMDAR hallmark properties of Mg^2+ ^block and Ca^2+ ^permeability were performed.

### The NR3B N and N+1 Site Amino Acids Are Crucial for Ca^2+ ^Permeability

The receptors' permeability for Ca^2+ ^ions is a characteristic chiefly dependent on the Q/R/N site residues of the constituent subunits. To test whether this also holds true for NR3-containing diheteromers, the ratio of Ca^2+ ^to monovalent cation permeability was determined. A key difference to be considered for NR1/NR2 and NR1/NR3 receptors is the agonist by which the diheteromer is activated. For the conventional NR1/NR2 combinations, it is mandatory to focus on glutamate/glycine-induced currents, as both agonists are required for full activation and govern the receptor's behavior *in vivo*. For NR1/NR3 diheteromers, it is necessary to consider solely glycine-evoked responses as a) NR1/NR3 form excitatory glycine receptors [15,21] and b) to minimize possible effects of *Xen*NR2B endogenous to the oocyte [[31], and compare [21]]. However, for all subunit combinations both glycine- and glutamate/glycine-induced responses were measured, and all reversal potentials and P_Ca_^2+^/P_mono _values are summarized in Table 2.

**Table 2:** Ca^2+ ^permeability of coexpressed mutant NMDAR subunits: Reversal potentials and P_Ca_^2+^/P_mono _ratios

	**E**_**rev(Gly) **_**[mV] (*n*)**	**P**_**Ca**_^**2+**^**/P**_**mono **_**± SEM (Gly) (*n*)**	**E**_**rev(Glu/Gly) **_**[mV] (*n*)**	**P**_**Ca**_^**2+**^**/P**_**mono **_**± SEM (Glu/Gly) (*n*)**
NR1-3a/NR2B	-23.1 ± 3.93 (*5*)	3.1 ± 0.65 (*5*)	-29.3 ± 1.79 (*5*)	2.3 ± 0.21 (*5*)

NR1-3a/NR2B(**G**N)	-15.3 ± 5.06 (*5*)	4.5 ± 1.02 (*5*)	-32.3 ± 2.73 (*5*)	2.0 ± 0.27 (*5*)

NR1-3a(**G**S)/NR2B	-39.7 ± 2.05 (*5*)	1.4 ± 0.13 (*5*)	-44.7 ± 2.20 (*5*)	1.1 ± 0.10 (*5*)

NR1-3a(**G**S)/NR2B(**G**N)	-46.8 ± 5.14 (*5*)	1.0 ± 0.18 (*5*)	-44.1 ± 1.24 (*5*)	1.1 ± 0.07 (*5*)

NR1-3a/NR3B	-68.4 ± 5.65 (*9*)	0.4 ± 0.11 (*9*)	-64.9 ± 4.26 (*8*)	0.5 ± 0.11 (*8*)

NR1-3a/NR3B(**N**R)	-34.7 ± 5.27 (*9*)	1.7 ± 0.61 (*9*)	-28.2 ± 1.71 (*8*)	2.5 ± 0.21 (*8*)

NR1-3a/NR3B(G**N**)	-49.2 ± 7.66 (*9*)	0.4 ± 0.31 (*9*)	-36.3 ± 2.75 (*8*)	1.7 ± 0.21 (*8*)

NR1-3a/NR3B(**NS**)	-46.8 ± 10.03 (*5*)	1.0 ± 0.57 (*5*)	-20.4 ± 4.74 (*4*)	2.5 ± 0.79 (*4*)

NR1-3a(**G**S)/NR3B	-64.5 ± 12.62 (*5*)	0.2 ± 0.20 (*5*)	-76.2 ± 7.92 (*5*)	0.1 ± 0.07 (*5*)

NR1-3a(**G**S)/NR3B(**N**R)	-83.1 ± 14.72 (*5*)	0.1 ± 0.13 (*5*)	-93.2 ± 10.33 (*5*)	0.1 ± 0.04 (*5*)

NR1-3a(**G**S)/NR3B(G**N**)	-54.6 ± 7.02 (*5*)	0.3 ± 0.11 (*5*)	-59.5 ± 7.61 (*5*)	0.3 ± 0.07 (*5*)

NR1-3a(**G**S)/NR3B(**NS**)	-38.0 ± 1.16 (*5*)	0.7 ± 0.03 (*5*)	-38.2 ± 2.74 (*5*)	0.7 ± 0.10 (*5*)

Overview of reversal potentials (E_rev_s) and calculated P_Ca_^2+^/P_mono _ratios of diheteromers featuring N and N+1 site mutant NMDAR subunits measured upon activation by either glycine alone (Gly, 10 μM) or Gly in addition to glutamate (Glu, 100 μM). Reversal potentials were measured in 8 mM Ca^2+ ^Ringer. Errors represent the standard error of the mean (SEM). n = number of cells.

For the conventional NR1/NR2 receptor, Ca^2+ ^permeability measurements after the application of glutamate/glycine gave a P_Ca_^2+^/P_mono _value of 2.3 for NR2B-containing receptors. For this conventional diheteromer, mutation of the NR1 N site to glycine approximately halved the relative Ca^2+ ^permeability to 1.1 for NR1-3a(GS)/NR2B. On the other hand, mutation of the NR2 subunit's N site residue to glycine had almost no effect in NR2B(GN)-containing receptors (P_Ca_^2+^/P_mono _= 2.0). For the combination of both mutated NR1 and NR2 subunits, P_Ca_^2+^/P_mono _values did not differ much from those obtained when only the NR1 subunit was mutated (P_Ca_^2+^/P_mono _= 1.1 for NR1-3a(GS)/NR2B(GN)).

**Figure 2:**
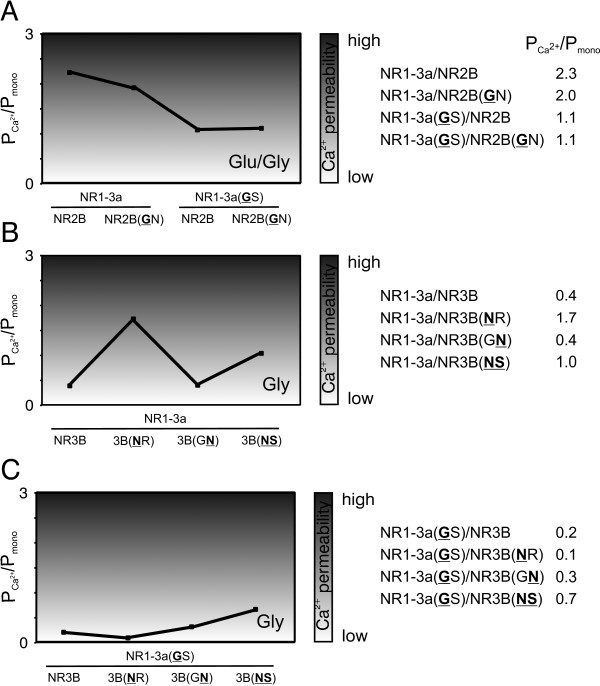
**Ca^2+ ^permeability of coexpressed mutant NMDAR subunits**. Overview of the Ca^2+ ^permeabilities of diheteromers featuring N and N+1 site mutant NMDAR subunits. To the right, P_Ca_^2+^/P_mono _ratios for the investigated combinations are given; the graphs represent those values on a relative scale. For NR1/NR2 combinations, measurements of Ca^2+ ^permeability were performed after the application of 100 μM glutamate (Glu) and 10 μM glycine (Gly). NR1/NR3 diheteromers were activated by glycine alone. n = 5-9.

Figure 2A illustrates this: Ca^2+ ^permeability is lowered if an NR3-like NR1-3a(GS) is present in the complex.

NR1/NR3 receptors behaved differently. Figure 2B depicts the changes in Ca^2+ ^permeabilities for NR1/NR3 receptors with mutations in one or both subunits' N or N+1 site residues.

It should be noted that for glycine-induced currents, the wild type NR1/NR3 diheteromer is not Ca^2+^-permeable, with a P_Ca_^2+^/P_mono _value of 0.4. Mutating the NR3B N site residue to asparagine increased the relative Ca^2+ ^permeability to 1.7, a value almost in the range of wild type NR1/NR2 receptors. On the other hand, replacing the NR3B N+1 residue by asparagine had no effect on Ca^2+ ^permeability upon glycine activation (P_Ca_^2+^/P_mono _= 0.4). If the receptor was activated by glutamate/glycine, Ca^2+ ^permeability was increased to P_Ca_^2+^/P_mono _= 1.7 (Table 2). However, in this case the potential influence of oocyte-endogenous *Xen*NR2B has to be taken into account. This endogenous subunit is capable of assembly with (exogenous) NR1, binds glutamate, and confers Mg^2+ ^sensitivity and Ca^2+ ^permeability to the resulting NR1/*Xen*NR2B receptor [31]. Such a subunit thus has the potential to distort results when measuring NR1/NR3 receptors in *Xenopus *oocytes [21], and needs to be taken into account when interpreting results obtained after application of glutamate.

For a receptor composed of both mutated subunits, Ca^2+ ^permeability is also slightly increased to P_Ca_^2+^/P_mono _= 1.0.

When the NR1 subunit carried a glycine at its N site, receptors were hardly Ca^2+ ^permeable, regardless of the NR3 subunit present (Figure 2C), with P_Ca_^2+^/P_mono _ranging from 0.1 to 0.7. Only for the double mutant combination of NR1-3a(GS)/NR3B(NS) a slightly increased P_Ca_^2+^/P_mono _ratio of 0.7 was recorded. However, if the NR1 subunit was also mutated, Ca^2+ ^permeability was never "rescued" to the same extent as when the mutation was only introduced in the NR3 subunit.

### The NR3B N and N+1 Site Amino Acids Are Crucial for Mg^2+ ^Sensitivity

The asparagines at the NR1 and NR2 N and N+1 sites have been demonstrated to be essential for Mg^2+ ^block of the receptor. Even though NR3 features a glycine and an arginine residue at the critical positions, it has been argued that these amino acids are not directly responsible for the lack of Mg^2+ ^sensitivity [7,8,16,32]. As those studies have exclusively investigated triple expression of NMDAR subunits, or diheteromers featuring the NR3A subunit, we examined the effect of the NR3B N and N+1 site amino acids in diheteromeric receptors.

**Figure 3:**
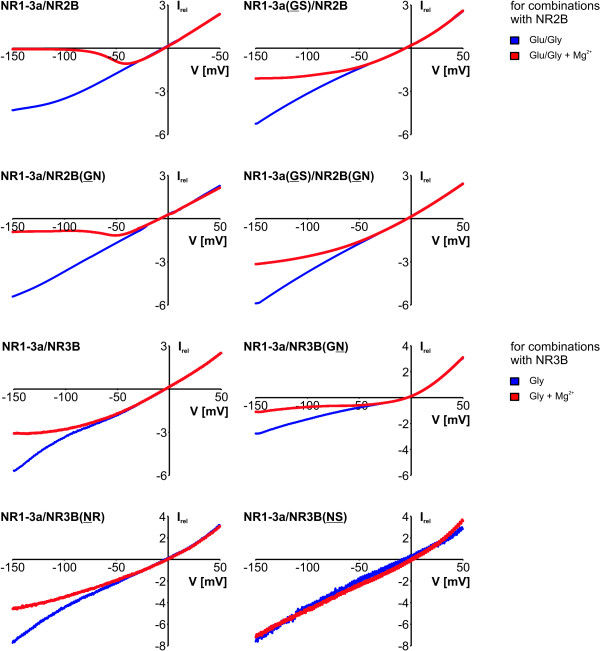
**Mg^2+ ^block of coexpressed mutant NMDAR subunits: IV relations**. IV relationships of the Mg^2+ ^block of N and N+1 site mutant NMDAR diheteromers. Shown are measurements between -150 mV and +50 mV after application of 10 μM glycine (Gly) alone or in coapplication with 100 μM glutamate (Glu) in the absence (blue traces) and presence (red bars) of Mg^2+^. Traces represent averages from 3-4 experiments per subunit combination (normalized to +20 mV).

The Mg^2+ ^block of either glycine- and glutamate/glycine-induced currents for all previously mentioned combinations was tested; values are summarized in Table 1. Shown in Figure 3 are representative current-voltage curves of the conventional NMDAR composed of NR1-3a/NR2B, and of NR1-3a/NR3B diheteromers, with either one or both subunits mutated to feature an NR3-like glycine at their N sites.

**Figure 4:**
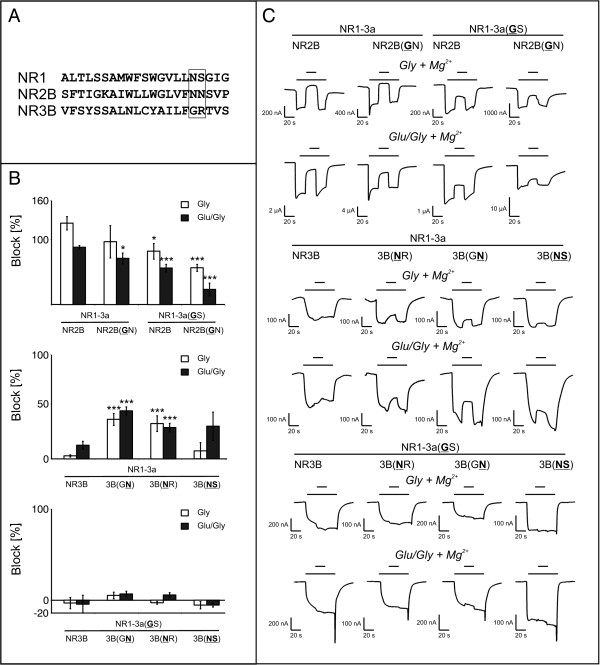
**Mg^2+ ^block of coexpressed mutant NMDAR subunits: mean values and current traces**. **A.** Amino acid sequence of the pore loop regions of rodent NR1, NR2B, and NR3B. The native amino acids at the N and N+1 positions are boxed. **B**. Mean values (± SEM) of the Mg^2+ ^block of N and N+1 site mutant NMDAR diheteromers. 0.5 mM Mg^2+ ^was applied after activation of the receptor with 10 μM glycine (Gly) alone or in coapplication with 100 μM glutamate (Glu). Statistical significance was determined relative to the respective wild type combination. n = 5-27, *p < 0.05; **p < 0.01; ***p < 0.005. **C**. Representative current traces of the Mg^2+ ^block of diheteromers featuring N and N+1 site mutant subunits. Agonists (conc. as in **B**) were applied for 60 s (long bar); Mg^2+ ^was added for 20 s during the application (denoted by the short bar).

If either subunit of the conventional NR1/NR2 receptor was equipped with this N site glycine, the full block by Mg^2+ ^was disrupted. Block of receptor currents still occurred in a voltage-dependent fashion below -50 mV, but in both cases only 70-80% of the glutamate/glycine-induced current response could be inhibited (Figure 4B, upper diagram). If both subunits featured a glycine at their N sites, only 25 ± 8.6% of the glutamate/glycine-induced responses were blocked by extracellular Mg^2+^. Figure 4C shows representative current responses of the aforementioned mutant receptors.

**Figure 5:**
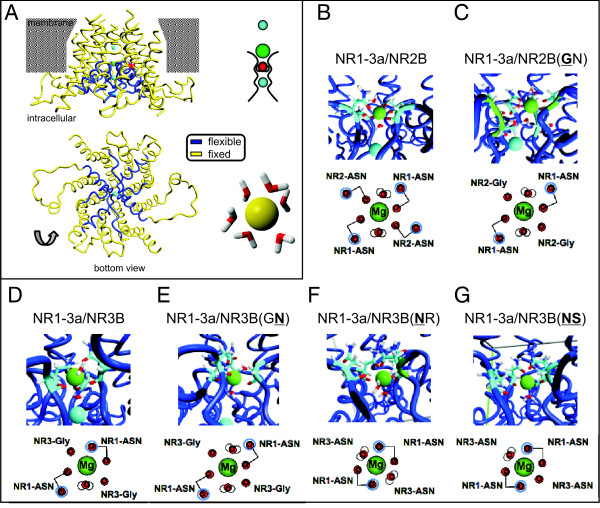
**Coordination of Mg^2+ ^within the pore of wild type and N and N+1 site mutant NMDARs**. Conformation of the pore region of an NR1/NR3 diheteromer. **A**. Side view of the selectivity filter of an NR1/NR3 diheteromer. Note that not the entire transmembrane region is shown. A similar conformation is employed by NR1/NR2 receptors. The N site is located at the tip of the pore loop; the N+1 site is located adjacent to that in the helix (marked by the red asterisk). Below, the same diheteromer is shown in a bottom view. The extensive protein loops belong to the NR1 subunits. To the right, a schematic representation of the selectivity filter of NMDARs is depicted: One Na^+ ^ion (blue ball) and one Mg^2+ ^ion (green ball) are coordinated in the pore with a water molecule (red) serving as a spacer. Bottom right: coordination of six water molecules by an Mg^2+ ^ion. **B.-G**. Coordination of Mg^2+ ^in NMDAR diheteromers. Mg^2+ ^is depicted as a green ball; the blue ball represents Na^+^. Below each structure is a schematic view of the coordination by water (red with white protons), side chain oxygens (red dots) and backbone oxygens (red dots with blue rings).

Figure 5A shows the conformation of the pore region of an NR1/NR3 diheteromer derived from a series of ns-scale molecular dynamics (MD) simulations. A similar conformation is employed by NR1/NR2 receptors. The N site residues are located at the tip of the pore loop, forming the narrow constriction. The Mg^2+ ^ion (green ball) is coordinated at this position, while the N+1 site amino acid is located in the pore helix (red star), contributing to the shape of the selectivity filter. Also shown in Figure 5A is a schematic representation of the ion permeation pathway of NMDARs. Coordination of Mg^2+ ^(green) by the N site residues is stabilized by a Na^+ ^ion (blue) inside the pore, with a water molecule (red) serving as a stabilizing spacer between the two. Above the pore region in the outer vestibule another sodium ion is localized. From this simulation it is plausible that the N site amino acid is directly involved in Mg^2+ ^coordination - and thus a key component for the Mg^2+ ^block. The N+1 site residue, located further behind the narrow constriction in the pore helix, shapes the selectivity filter, but cannot be solely responsible for ion coordination. Additionally, water is required for the coordination of Mg^2+ ^ions. Simulations were performed in a water-filled box, keeping the outer TMD regions rigid and allowing flexibility for the pore helices and the selectivity filter. Consistent with this, in the absence of water molecules (*in vacuo *simulations), stable coordination of Mg^2+ ^cannot be achieved. In order to get an idea how stable Mg^2+ ^coordination can be obtained, we studied Mg^2+ ^coordination in a water-filled box in an MD simulation. Mg^2+ ^is coordinated by 6 H_2_O molecules forming an octaeder-shaped water shell. An octahedron-shaped coordination pattern for Mg^2+ ^ions is also seen in Mg^2+ ^and ATP/GTP binding proteins and suggests a favored and stable conformation (Figure 5A, lower right). NMDAR residues may contribute Mg^2+^-coordinating site components via their backbone carboxygens or by their side chain oxygens. In our simulations, there was always an octahedron-like Mg^2+ ^coordination pattern observed (Figure 5B-G). However, water molecules contribute to different degrees to the Mg^2+ ^coordination as well (Figure 5B-G).

As described, the Mg^2+ ^block of NR1/NR2B receptors was significantly reduced when either subunit's N site asparagine was mutated to the NR3-like glycine. The MD simulations of the NR1/NR2B(GN) mutant supports the electrophysiological findings by revealing the impaired coordination of the Mg^2+ ^at the pore tip for these mutant receptors:

Figure 5B details the coordination of an Mg^2+ ^ion in a conventional wild type NR1/NR2 receptor: Four backbone oxygens (red balls) coordinate the ion in the presence of two water molecules. The oxygens contributed by the asparagine side chains (in blue rings) stabilize this conformation. If the NR2 N site asparagine is replaced by glycine, coordination of Mg^2+ ^via backbone oxygens is still possible, but the conformation lacks two stabilizing asparagine oxygens (Figure 5C).

In NR1/NR3 diheteromers the opposite effect was seen: While the wild type is not blocked by Mg^2+ ^ions, the exchange of either NR3's N site glycine or its N+1 site arginine to asparagine rendered the diheteromer Mg^2+^-sensitive. However, this effect was more pronounced if the N+1 site arginine was exchanged. Representative current-voltage relationships are shown in Figure 4C.

Figure 5D shows the state of a wild type NR1/NR3 pore after an MD simulation. To supply six coordination sites for one Mg^2+ ^ion, one NR1 asparagine residue oxygen would have to substitute for a water molecule. This conformation, which exerts constraints on the pore structure, might be rather unstable, explaining why NR1/NR3 receptors are not blocked by Mg^2+ ^ions. Furthermore, most of the transmembrane domains flanking the pore helices are locked in an artificial, rigid state in the MD simulations. In a fully flexible receptor *in vivo*, such an asymmetric conformation of the pore might not be supported.

Mutating the NR3 N+1 arginine to asparagine results in the same amino acid combination of glycine-asparagine as in mutant NR2 subunits, where the N site asparagine is mutated to glycine. When coexpressed with NR1, both mutants assemble into receptors that are slightly blockable by Mg^2+^. When comparing Figure 5E to Figure 5C, it is striking how similar the mode of Mg^2+ ^coordination is in those two receptors that are very different except for their N and N+1 site amino acids. This accordance in Mg^2+ ^coordination, solely dependent on the N and N+1 site residues, supports the similarity of those receptors regarding the Mg^2+ ^block.

Mutating the N site glycine of NR3 to asparagine leads to the formation of NR1/NR3 receptors that are blockable by Mg^2+ ^to a similar extent as when the N+1 site residue of NR3 is mutated - the mechanism might be rather different, though. As Figure 5F shows, Mg^2+ ^is tightly coordinated in an NR1/NR3B(NR) diheteromer. However, the stabilizing sodium ion within the permeation pathway is not supported by such a conformation, and leaves the pore. The position of this Na^+ ^ion is critically dependent on the N+1 residue. Its leaving the pore might cause a destabilizing effect counteracting the tight Mg^2+ ^coordination, and resulting in a net effect not unlike that of NR1/NR3B(GN) diheteromers.

If both the N site glycine and the N+1 site arginine were exchanged for NR1-typical asparagine and serine residues, no block could be detected upon application of extracellular Mg^2+^. The middle diagram in Figure 4B summarizes the mean values measured for the individual combinations, representative current traces are depicted in Figure 4C.

As Figure 5G shows, the lack of Mg^2+ ^sensitivity is likely not due to the failure to coordinate Mg^2+^. In fact, comparing the MD simulations of the pore regions of NR1/NR3B(NR) and NR1/NR3B(NS) receptors (Figure 5F and 5G, respectively), the coordination of the Mg^2+ ^ion in principle appears very similar between the two. There is a notable difference, though: In the NR1/NR3B(NR) diheteromer, both NR1 N site asparagines supply two oxygens (one from the backbone and one from the side chain, blue encircled) for coordination of Mg^2+^. The other coordination sites might be occupied by water molecules. In contrast, the Mg^2+ ^ion in the NR1/NR3B(NS) pore is - according to the MD simulation - asymmetrically coordinated, with only one NR3 asparagine involved. Whether a fully flexible receptor in its native environment would support an asymmetrical arrangement of its pore is questionable. The only other subunit for which an MD simulation predicts a pore conformation that does not have a two- or fourfold rotational symmetry is the NR1/NR3B wild type diheteromer - which is not coordinating Mg^2+ ^under natural conditions.

As it was generally possible to partly rescue Mg^2+ ^sensitivity by mutating the NR3 N or N+1

residues, those mutants were coexpressed with an NR1 subunit equipped with the NR3-like glycine at the N position. As shown in the lower diagram of Figure 4B, none of the receptor compositions featuring NR1-3a(GS) were blockable by Mg^2+^. In fact, if the NR1-3a(GS) mutant was coexpressed with either the wild type NR3B or the double mutant NR3B(NS), currents appeared to be potentiated (if feebly) by the addition of Mg^2+ ^ions. However, mean values for NR1-3a(GS)-containing receptors were not different from the NR1/NR3B wild type receptors in a statistically significant way. Representative current traces are depicted in Figure 4C.

It is noteworthy that for receptors featuring the mutant NR1-3a(GS) subunit, current traces showed a distinct off-peak (Figure 4C). A similar rebound effect has been described by Madry and colleagues [33], who could show this effect to be due to the release of the NR1/NR3 receptor from NR1-mediated desensitization. A similar effect might be caused or enhanced by using the mutant NR1-3a(GS) subunit in combination with NR3B.

## Discussion

The influence of the amino acids at the pore's narrow constriction - the so-called N and N+1 sites - on hallmark NMDAR characteristics has been recognized early on. Since shortly after the cloning of the NR1 subunit [4], the groundbreaking work on the importance of the NMDAR N site has been conducted using a combination of site-directed mutagenesis, heterologous expression, and electrophysiological characterization of the mutants. NMDARs featuring NR1 subunits with AMPAR-typical glutamine or arginine residues at their N sites showed a marked reduction of block by extracellular Mg^2+^. Likewise, Ca^2+ ^permeability was reported to be reduced [2].

Similarly, Wollmuth and colleagues could show that exchanging either the NR1 or the NR2 N site residue to glutamine effectively increases the diameter of the pore's narrow constriction [6]. The increased pore diameter led to a decrease in Ca^2+ ^permeability [6] - a finding suggestive for NR3B, which natively carries glycine at its N position.

Prompted by these findings, the NR3 subunits' unique N and N+1 site amino acid combination has been examined with regard to functional impact almost immediately after the discovery of this "NMDAR-like" [8] subunit. While Ciabarra and colleagues investigated an NR3A with an AMPAR-typical glutamine in lieu of the native N+1 site arginine [7], Sucher and colleagues generated an NR3A featuring asparagine at the N+1 position [8]. In a triple coexpression with NR1 and NR2, no influence of the mutated NR3A(GN) on Mg^2+ ^block could be determined [8], nor did the NR3A(GQ) subunit form functional homomers [7].

Yamakura and colleagues investigated the newly-found NR3B subunit shortly after its discovery, and equipped it with the two adjacent asparagines found at the N and N+1 sites of NR2, but reported no influence of the mutant NR3 on the block of NMDARs by Mg^2+ ^or APV [16].

In this context it is noteworthy that a recent study of fluorescence-labeled NMDAR subunits failed to show triheteromeric assembly of NR1, NR2, and NR3 subunits in *Xenopus *oocytes [34]. Instead, Ulbrich and Isacoff postulate the existence of separate NR1/NR2 and NR1/NR3 receptor populations in the oocyte membrane [34]. In this case, effects of NR3 might have been masked by the presence of conventional NR1/NR2 receptors in previous studies. We therefore consider it a valid question whether the NR3 N and N+1 site residues are really not at the core of reduced Mg^2+ ^block and Ca^2+ ^permeability in NR3-containing receptors.

The success of previous studies to investigate functional N and N+1 site mutants prompted the construction of a new set of point mutants for investigation in diheteromeric expression. However, some deviations from the mutants described previously were incorporated in the design: The NMDAR-typical asparagine was introduced in place of the native NR3 N site glycine. However, in wild type NR3B, the small N site glycine residue might not contribute much as a structural determinant. It is conceivable that the much larger arginine at the NR3 N+1 site protrudes into what constitutes NR3's contribution to the N site, and is really at the core of the low Ca^2+ ^permeability. An arginine at the N position is reminiscent of the GluR2 subunit, where editing glutamine to arginine at this site virtually abolishes the subunit's Ca^2+ ^permeability. Therefore, the NR3 N+1 site was mutated to asparagine independently, producing an NR3B mutant equivalent to the NR3A(GN) examined by Sucher and colleagues [8].

To examine a full N and N+1 site mutant NR3, the approach by Yamakura and colleagues to use adjacent asparagines was not followed [16]. As NR3 is much more closely related to NR1 than to NR2, considering their amino acid sequence (and thus, ligand binding capability), the NR3B subunit was equipped with the NR1-like combination of asparagine and serine.

The diheteromers investigated shed some light on crucial questions of the NR3B structure-function relationship, and the differences between this subunit and its closest relative, NR1.

### N and N+1 Site Mutants Assemble Functionally in a Heterologous Expression System

Previous studies on conventional NR1/NR2 N site mutants were successful in reporting assembly and function of the mutant subunits. This is not surprising, as neither the initial contact-forming domains, nor the dimerization interface lie within the depth of the pore [35].

As expected, the finding that NR1 and NR2 N site mutant subunits can functionally assemble into NMDARs could be corroborated. While affinities where not measured quantitatively, at least qualitatively the agonist profiles of conventional NR1/NR2 NMDARs and NR1/NR3 glycine receptors were not altered by introducing mutations: The conventional NMDARs required glutamate for full activation, while all NR1/NR3 diheteromers were fully activated by glycine. Slight potentiation of NR1/NR3 responses by additional glutamate can be attributed to the influence of endogenous *Xen*NR2B, the presence of which could not be ruled out for all measurements [[31], and compare [21]]. For uncompromised investigations of the NR1/NR3 diheteromers' properties, it is therefore sensible to consider Mg^2+ ^block and Ca^2+ ^permeability measurements after the application of glycine only.

While the agonist profile was generally unaltered, it is noteworthy that NR1/NR2B receptors gave rise to the lowest current responses. If either the NR1 or NR2 subunit was equipped with an NR3-like glycine at its N site, amplitudes were increased. This effect was increased if the NR1 subunit carried the mutation, and was strongest if both subunits were mutated - roughly approaching the sum of the effects of the single mutations. Considering the aforementioned study by Wollmuth and colleagues [6] on similar mutants of NR1 and NR2A, increased current amplitudes might simply be caused by widening the narrow constriction of the receptor pore: Enlarging the pore diameter thus effects a higher conductivity of the channel, an effect that was reported larger if the NR1 subunit carried the mutation, and additive if both subunits did [6]. If this line of thought is followed, the observed increase in amplitudes might already hint at a lowered Ca^2+ ^permeability and Mg^2+ ^block, as coordination sites for divalent cations are lost in the widened pore.

Current responses of NR1/NR3 diheteromers were similarly dependent on the N and N+1 site residues: The wild type glycine receptor again gave rise to the lowest current responses, but neither of the receptors featuring the NR3B(NR) or NR3B(NS) mutants differed much from the wild type in their current amplitudes. Replacing the bulky arginine at the NR3 N+1 position with the slightly smaller asparagine in NR3B(GN) might also increase the diameter of the permeation pathway by simple steric effects, explaining why the largest current responses were elicited from receptors with this mutant. The fact that in AMPARs an arginine at the Q/R site abolishes Ca^2+ ^permeability, together with the notion that in a possibly widened NR1/NR3(GN) pore Ca^2+ ^coordination might be disrupted, suggests that the N+1 arginine in NR3B is crucial for Ca^2+ ^permeability.

### The NR3B N and N+1 Site Amino Acids Are Crucial for Ca^2+ ^Permeability

Ca^2+ ^permeability - or the lack thereof - might well be the single most important functional property impacted by NR3. Ca^2+ ^as a second messenger triggers a number of intracellular signaling cascades, among them those effecting the incorporation of new NMDARs into the membrane and supporting LTP. It is therefore crucial to understand how NR3 downregulates this most essential property of NMDARs.

The arginine at the NR3 N+1 site would be a likely candidate for lowered Ca^2+ ^permeability. Like the arginine in edited GluR2, this residue might effect electrostatic hindrance and the disruption of Ca^2+ ^hopping relays. Equipping NR1 with an arginine at the N site reduces Ca^2+ ^permeability [2]. Here we show, however, that NR3B can be made Ca^2+ ^permeable by exchanging one amino acid, but that is not the arginine at the N+1 site.

In fact, the NR3B(GN) mutant is no more Ca^2+ ^permeable than its wild type counterpart. Even the double mutant NR3B(NS), which carries both N and N+1 site residues of NR1, displays only a modest increase in Ca^2+ ^permeability. But exchanging the NR3 N site glycine for asparagine yields a subunit that assembles into a glycine receptor more permeable for Ca^2+ ^than NR1/NR3 wild types.

In contrast, if the NR1 subunit carries an NR3-like glycine at its N position, Ca^2+ ^permeability is non-existent, regardless of how the NR3 N site is equipped. This is also true if the mutant NR1(GS) subunit is coexpressed with NR2B: as a result of the glycine mutation, Ca^2+ ^permeability is lowered. The same mutation in NR2 subunit has less of an effect. This suggests a cooperative effect: a glycine residue at the N site of either the NR1 or the NR3 subunit is sufficient to compromise Ca^2+ ^permeability. NR3 naturally supplies this glycine, and thus downregulates NMDAR-mediated Ca^2+ ^influx - as has already been shown for proposedly triheteromeric NMDARs [36,37]. On the other hand, possibly due to the unique alignment of the NR1 and NR3 residues at the level of the pore [32], an arginine at the NR3 N+1 position does not impede permeability for Ca^2+^.

### The NR3B N and N+1 Site Amino Acids Are Crucial for Mg^2+ ^Sensitivity

While the diameter of the pore is crucial for ion permeation and Ca^2+ ^coordination, pore size has been shown to be a minor determinant of how effectively Mg^2+ ^blocks conventional NMDARs [3]. Rather, the presence of Mg^2+^-coordinating residues is crucial for a full block of the receptor at negative membrane potentials.

From the investigation of N site NR1 and NR2 mutants, it can be concluded that the full block by Mg^2+ ^is compromised and significantly reduced if either one subunit in an NR1/NR2B receptor carries an NR3-like glycine at its N site. The block is reduced even further if both subunits are mutated; the mutations appear to be additive.

Curiously, for currents elicited by the sole application of glycine, the combination of wild-typic NR1-3a/NR2B subunits appeared to be blockable by Mg^2+ ^to an extent greater than 100%. To a lesser extent, this was also observed for NR1-3a/NR2B(**G** N) receptors. (Figure 4B). Obviously, if Mg^2+ ^blocks more than the induced current, part of the block must stem from the inhibition of leak currents (LC). If the agonist-induced current (IC) is comparatively small, the ratio LC/IC becomes relatively large. On the other hand, if the induced current becomes large (as is the case for the coapplication of glutamate/glycine), the LC/IC ratio decreases. Given the experimental setup of the two-electrode voltage clamp method, a small LC/IC value would not cause a measurable change in block, whereas for a large LC/IC ratio, the contribution of the leak current (or block thereof) might be detected in recordings.

In line with this, the wild-typic NR1-3a/NR2B receptors (where current block appeared to be more than 100%) gave the smallest current responses upon the sole application of glycine compared to the mutant NR1/NR2 subunit combinations.

Further supporting this interpretation, for NR1/NR3 receptors, which are not or less sensitive to Mg^2+ ^(depending on the mutants present), no such effect could be seen.

It is noteworthy that the extent of Mg^2+ ^block differs depending on the agonist applied. This finding strengthens the prevailing view of an unequal contribution of NR1 and NR2 to the Mg^2+ ^block, but shifts emphasis to the NR1 N site residue. Equipping this closest relative of NR3 with an NR3-like glycine reduces the NMDARs sensitivity to Mg^2+^.

As NR2B-containing receptors can be made (partially) insensitive to Mg^2+ ^by either equipping them with an NR3-like N site residue, or by coexpressing them with a mutated NR1, it was not surprising that NR1/NR3 diheteromers could be made Mg^2+ ^sensitive by the reverse mutation.

Generally, when measuring the Mg^2+ ^block of NR3-containing diheteromers, glycine-induced currents should be considered rather than glutamate/glycine-induced responses, which are usually influenced by endogenous *Xen*NR2B and tend to display more "conventional" properties. But considering the glycine-evoked responses, it is obvious that if either NR3's N or N+1 site residue is mutated to asparagine, coordination of Mg^2+ ^is possible to an extent that markedly blocks the receptor at -70 mV. The mechanism by which this is achieved, however, might differ for the two mutants.

Generally, assembly of NR1 and NR3 might be critically different from that of NR1 and NR2, as shown by SCAM studies: It has been proposed that NR1 and NR3A assembly brings a ring of threonine residues in the outer channel verstibule into a planar alignment [32]. As the TMD B of the NR3 subunit is rigid during gating, the threonine ring constitutes a constriction located externally of the conventional narrow constriction [32]. According to this study, the NR3A N site is not involved in the selectivity filter of the NR1/NR3 diheteromer [32]. If one adopts this finding for the NR3B subunit, it is obvious that the general view of an "inactive" NR3 N site has to be reconsidered in the light of the data presented here.

With the combination of two rather large residues in the NR3B(NR) mutant, or the asparagine at the N+1 position in NR3B(GN), Mg^2+ ^ions can be coordinated well enough to block the receptor, and more efficiently if the asparagine is located at the N+1 site. In summary, the picture forms of a cooperative Mg^2+ ^coordination between (conventionally) NR1 and NR2, but also NR1 and NR3 (given the appropriate residue is supplied at the N+1 position). This also explains why the combination of only NR1-like N and N+1 amino acids in the NR1/NR3B(NS) diheteromer does not support an Mg^2+ ^block. Consequently, equipping the NR1 subunits with a glycine at its N site should also abolish the block, which indeed is the case: There is no NR1 N site asparagine to interact with the residue at the N+1 position of the partner subunit; consequently, the prerequisites for Mg^2+ ^coordination are not met.

In summary, contrary to previous studies, the present experiments suggest that both the NR3 N and N+1 site residues are responsible for the (lack of) sensitivity to Mg^2+ ^by interacting with the NR1 N site asparagine to coordinate the ions.

## Conclusions

In this study, we provide new insight into the structure-function relationship of NR3B, the least well characterized member of the NMDAR subfamily. While the importance of the N and N+1 site amino acids of NR1 and NR2 subunits for Mg^2+ ^block and Ca^2+ ^permeability have been thoroughly established, the equivalent residues in NR3 have been attributed little functional impact. The NR3B subunit supported both Ca^2+ ^permeability and Mg^2+ ^sensitivity when equipped with an NR1-like N site residue; conversely, both NR1 and NR2 subunits could be made Mg^2+ ^insensitive and less Ca^2+ ^permeable by replacing their N site amino acids with the NR3-like glycine. Thus, we provide evidence that the NR3 N and N+1 sites are critical determinants for the unique NR3-mediated functional traits of Mg^2+ ^insensitivity and Ca^2+ ^impermeability.

## Authors' contributions

NAC and GH generated mutant receptor subunits. NAC and AO carried out the electrophysiological measurements. NAC performed the statistical analyses, and drafted the manuscript. GS performed the MD simulations and participated in writing the manuscript. MH participated in the design and supervision of the study and writing of the manuscript. All authors read and approved the final manuscript.
